# Evidence for funerary behaviour and lethal trauma in a new Neanderthal individual from Shanidar Cave

**DOI:** 10.1038/s41598-026-69193-z

**Published:** 2026-10-15

**Authors:** Emma Pomeroy, Christopher O. Hunt, Lucía López-Polín, Nohemi Sala, Ana Pantoja Pérez, James Holman, Jessica Twyman, Paul Bennett, Tim Reynolds, Ross Lane, Duncan Pirrie, Graeme Barker

**Affiliations:** 1 Department of Archaeology, University of Cambridge, Henry Wellcome Building, Cambridge, CB2 1QH UK; 2 School of Biological and Environmental Sciences, Liverpool John Moores University, Liverpool, UK; 3 McDonald Institute for Archaeological Research, University of Cambridge, Cambridge, UK; 4 Institut Català de Paleoecologia Humana i Evolució Social, Tarragona, Spain; 5 Universitat Rovira i Virgili, Tarragona, Spain; 6 Centro Nacional de Investigación sobre la Evolución Humana, Burgos, Spain; 7 Canterbury Archaeological Trust, Canterbury, UK; 8 Birkbeck, University of London, London, UK; 9 Helford Geoscience LLP, Helston, UK

**Keywords:** Taphonomy, Morphology, Wood technology, Care, Facial trauma, Anatomy, Evolution

## Abstract

Shanidar Cave has long featured in debates concerning Neanderthal cognitive capacity and behaviour, including evidence for care and intentional burial. By identifying additional individuals, including Shanidar A reported here, new excavations have expanded a burial cluster first identified in 1960. Shanidar A, an older adult, was deposited around 75,000 years ago, decayed, and their skeletal remains were subsequently disturbed by a flash flood. Our findings raise the minimum number of individuals in this unparalleled cluster to five, supporting interpretations of complex, potentially meaningful Neanderthal mortuary behaviour. Shanidar A also demonstrates major non-fatal trauma to the face and rare evidence of perimortem penetrating trauma among Neanderthals. Trauma among Middle and Upper Palaeolithic Neanderthals and modern humans, and subsequent survival, is common. However, the facial injury was severe and unusual. It suggests care from group members, the evolutionary origins of which are also contentious. The nature of the perimortem penetrating traumatic injury adds to a small corpus of such injuries in the hominin fossil record. The morphology of this scapula perforation is consistent with injury from a wooden spear, providing valuable (albeit indirect) evidence for the use of wooden implements in the Middle Palaeolithic, which is often difficult to demonstrate given the rare preservation of organic materials.

**Supplementary Information:**

The online version contains supplementary material available at https://doi.org/10.1038/s41598-026-69193-z.

## Introduction

The evolutionary origins of intentional treatment of the dead, and its implications regarding capacities for abstract thought and caring behaviour in the hominin lineage, continue to be a matter of contention^1–4^. Evidence for such behaviour amongst Neanderthals has received particular attention as part of wider discussions regarding their behaviour and cognitive capacity^1,5–7^. Although mortuary treatment of the dead among Neanderthals has become widely (though not universally) accepted on a general level, whether individual cases represent intentional mortuary treatment is still debated^8–12^, and whether emotional attachment, practicality and/or symbolic meaning underpinned this behaviour is challenging to discern (e.g.,^6,13^).

**Fig. 1:**
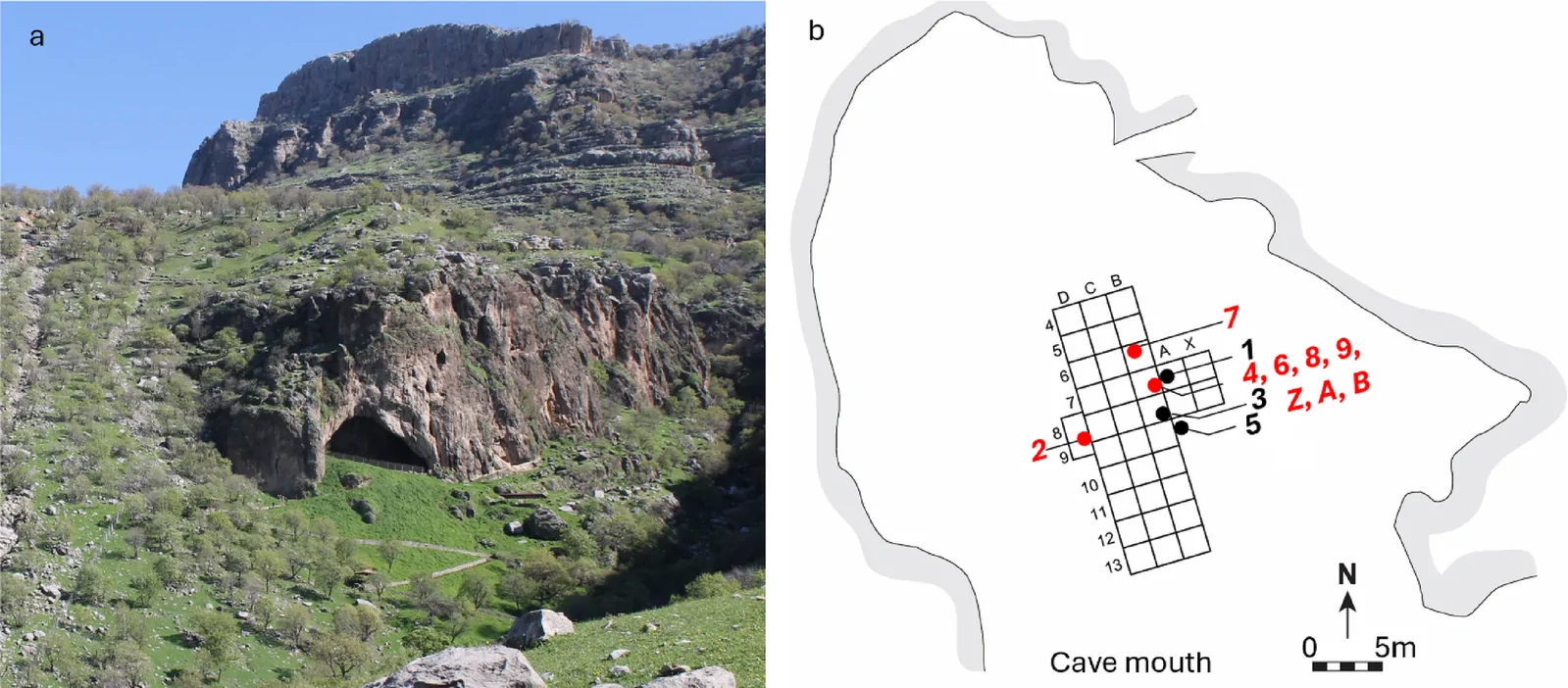
Shanidar Cave. **(a)** View of Shanidar Cave from approximately the south. (Photo: G. Barker). **(b)** Plan of Shanidar Cave, showing Ralph Solecki’s excavation grid, with the locations of the Neanderthal remains indicated according to their assigned identification numbers e.g. 1= Shanidar 1. Black text denotes individuals dating to > 50 ka, while red denotes individuals dated to ~ 70–75 ka. Image: R. Lane and E. Pomeroy.

The tight cluster of individuals associated with the Neanderthal skeletal remains designated Shanidar 4 (the famous ‘flower burial’) from Shanidar Cave, Iraqi Kurdistan (Fig. 1), may provide some of the clearest evidence of symbolic mortuary (i.e., funerary) behaviour among Neanderthals. The cluster comprises the skeletal remains of at least four Neanderthal individuals discovered by Ralph Solecki’s team in 1960 and designated by him Shanidar 4, 6 and 8 (adults) and Shanidar 9 (an infant). During new excavations that began in 2015, the skeletal remains of at least three adult individuals have been recovered directly adjacent to the 1960 individuals and designated Shanidar Z, A and B^14,15^. The remains of Shanidar Z and B, and possibly A, would have been truncated by the removal of the 1960 remains in a single sediment block^16^.

**Fig. 2:**
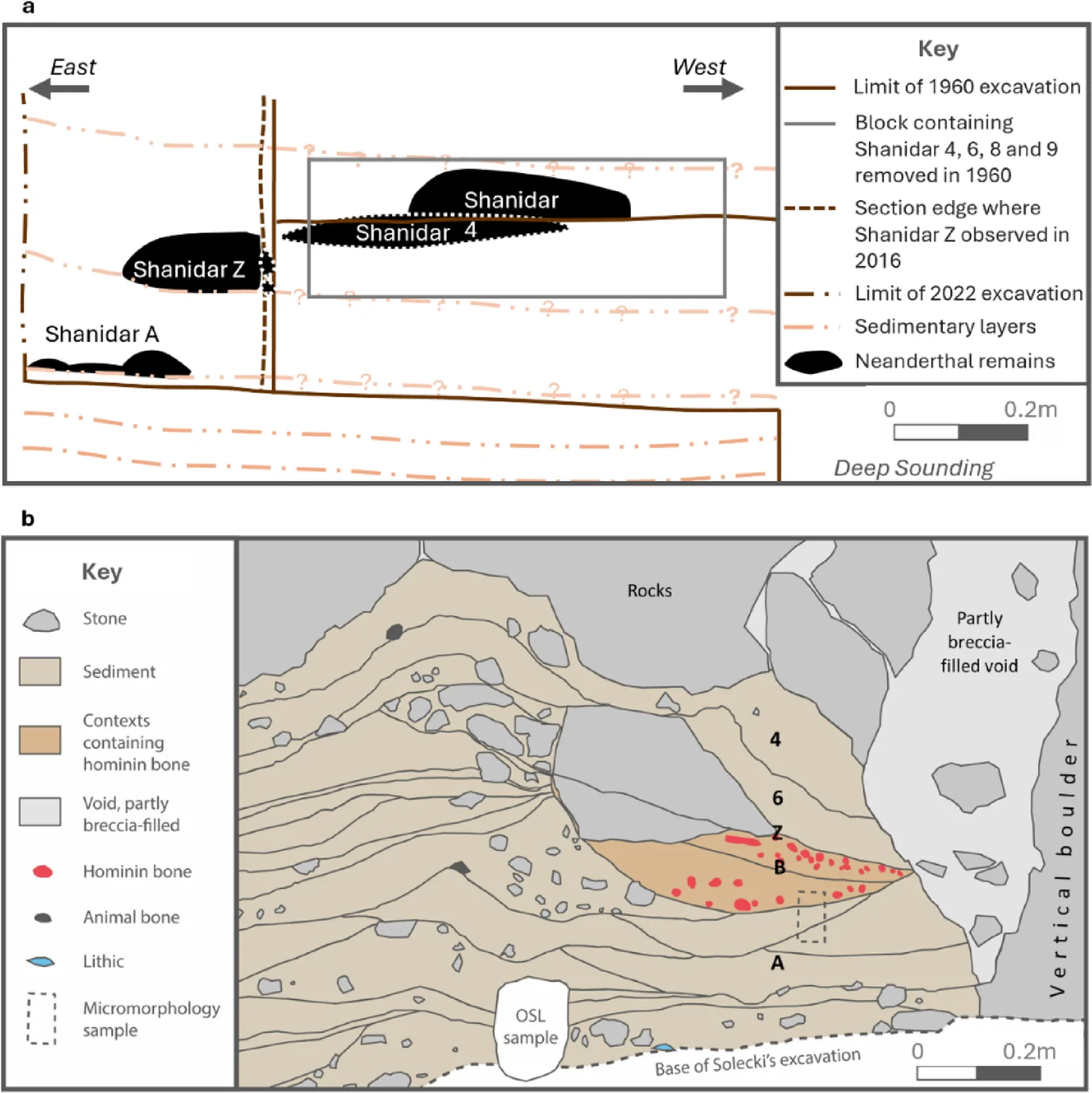
**(a)** Schematic diagram looking approximately south depicting the spatial relationships between the Neanderthal individuals in the Shanidar 4/Z cluster. Sediments around the skeletal remains are mostly waterlain, and further boulders from later rock fall (depicted in **(b)**) overlay these sediments. The skeletal elements designated Shanidar 8 by Solecki are not shown as their precise location is unclear. The large vertical boulder (see 2b) lies directly behind (to the south of) the remains illustrated. Image: E. Pomeroy. (b) Section drawing looking approximately east showing the spatial relationships between individuals in the Shanidar 4/Z cluster. The level of the uppermost remains of each individual is indicated by the relevant number/letter (e.g., 6 = Shanidar 6, A = Shanidar A, etc.). Shanidar Z and B are shown in situ (red). The Shanidar 4 and 6 remains would have been towards the viewer (i.e. further west); their heights are estimated from Solecki’s publications and archival records (see^16^). The Shanidar A remains were further away from the viewer i.e., to the east of the section depicted here, and due to the slope of the sediments from east to west, in fact lay in contexts (stratigraphic units) just above the level of the base of Solecki’s excavation. The overlying rocks and the large vertical boulder to the right would have projected above the contemporary surface of the cave floor. The depressions below Shanidar Z/B, including that in which the Shanidar A remains lie, are additional shallow water channels resulting from separate flash flooding events. Image: E. Pomeroy and C. Hunt.

All the individuals from the Shanidar 4/Z cluster date to ~ 70–75 ka (thousand years ago, see^14^), and lay within an area approximately 1.5 m long, 1 m wide and 1 m deep. Relative to the cave mouth, the remains are located directly behind a large vertical boulder that had fallen from a geological fault in the cave roof, and which would have been a prominent marker above the contemporary cave floor^16^ (Fig. 2). The field evidence, including sediment micromorphological analysis, indicates the excavation of a shallow depression for the placement of Shanidar Z^14^, and multiple deposition events of Neanderthal remains^16^. Although the heavily criticised^13,17^‘flower burial’ theory proposed by Solecki^7,18^ and Leroi-Gourhan^19^ has not stood up to further scrutiny^20^, the repeated use of a single circumscribed space to deposit the dead within a relatively short time-span (likely within generations, given that sequential deposits were in most cases made before full decomposition of the previously deposited body) is difficult to explain except in terms of intentional patterned behaviour. The highly restricted area within which the cluster was deposited points to specific meaning being attached to this location, rather than pure practicality or sentiment.

The argument that these individuals represent a single event where a group huddled behind the vertical rock for protection from the cold can be excluded. First, it is clear that the deposition of individuals occurred in multiple separate events, since there is a sedimentary separation of different individuals^16^, and some were disturbed by the deposition of the chronologically younger individual(s)^8,18^. Secondly, the sedimentary contexts indicate deposition in periods of mild climate, based on palynology and sedimentology, making death from exposure unlikely^14^. Therefore, the most parsimonious explanation for the number of individuals deposited in this compact space on several separate occasions is intentional activity.

Pomeroy et al.^14^ presented an overview of the Shanidar Z remains and their archaeological context. The purpose of the present study is to give an overview of the skeletal remains attributed to Shanidar A, their taphonomy, sedimentary context, and their relationship with the rest of the Shanidar 4/Z cluster, along with evidence for both antemortem and perimortem physical trauma.

## Results

### Shanidar A

**Fig. 3:**
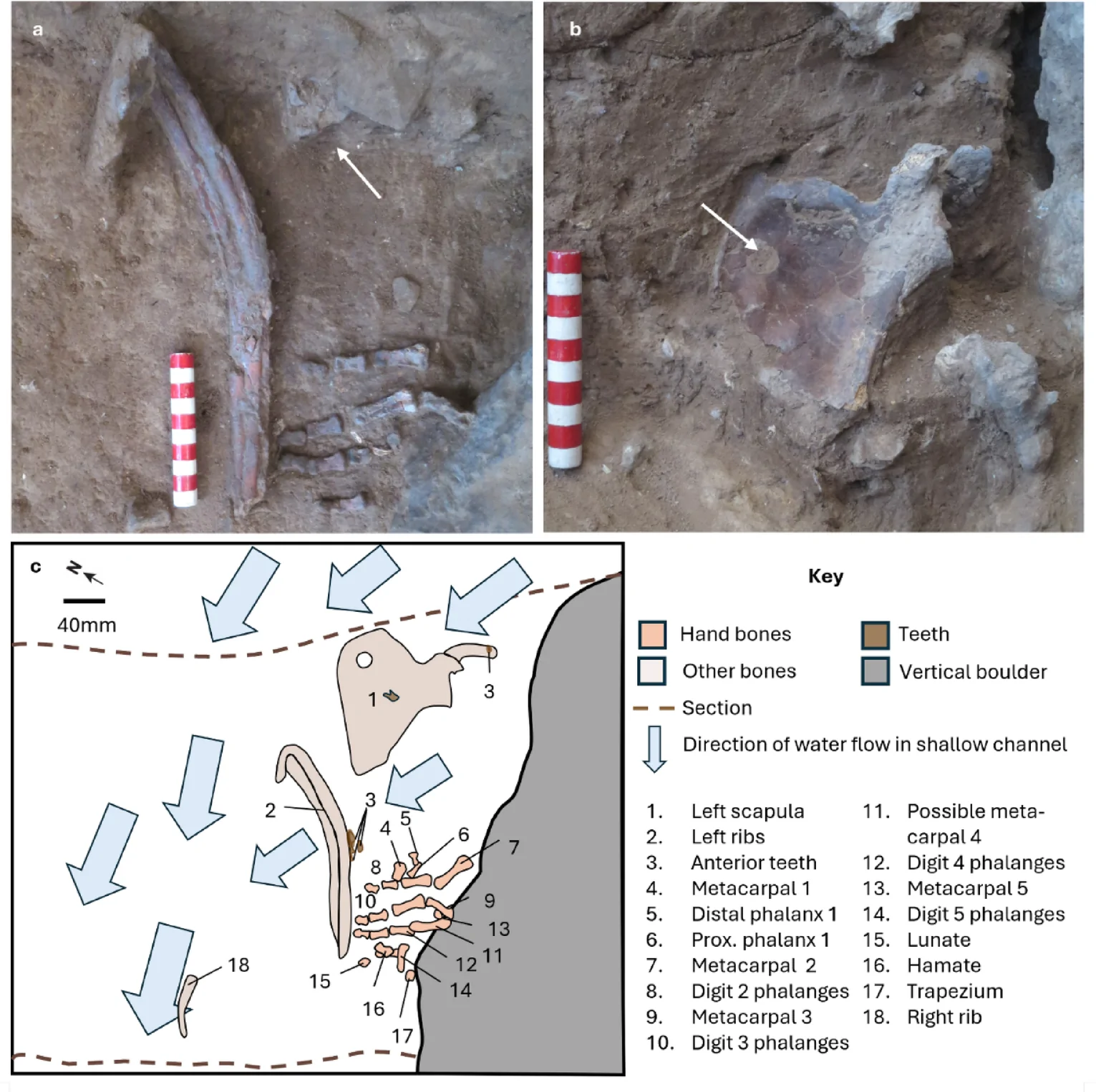
The Shanidar A remains *in situ*. **(a)** The left ribs and right manual phalanges. The white arrow indicates the inferior angle of the left scapula. Scale: 100 mm in 10 mm divisions. **(b)** The left scapula. The inferior angle was removed previously when it was initially exposed in the section, and the remainder of the scapula (as shown in the photograph) was exposed as the section was cut back. The perforation of the scapula discussed in the text is clearly visible, and indicated with a white arrow. Scale: 100 mm in 10 mm divisions. **(c)** Schematic scale plan of the Shanidar A remains based on *in situ *photographs. The waterlain sediments in which the bones lay were deposited by shallow flows moving in the direction indicated by the blue arrows. Images: G. Barker, E. Pomeroy and C.O. Hunt.

The Shanidar A skeletal remains lay in a waterlain sedimentary context, gently sloping from east to west (Figs. 2b and 3c) approximately 150 mm below the thorax of Shanidar Z (Fig. 2)^14^ and were excavated in 2019 and 2022^15,16^. Therefore, unlike the other individuals from this cluster, whose remains (as far as can be discerned) formed a group that overlapped or lay vertically adjacent to one another, the Shanidar A remains were clearly separated in vertical space from the rest of the cluster. However, the close proximity and positioning of the remains with a broadly similar orientation to (head to the east, reclining on back) and directly below Shanidar Z suggest that Shanidar A could be part of this same group.

An inventory of skeletal and dental elements attributed to Shanidar A is given in Table S1. The remains comprise a largely complete right hand, two left ribs, a right rib fragment, a left scapula and seven teeth. We attribute all the skeletal remains and six of the teeth to a single individual, Shanidar A, on the basis that: (1) there are no duplicated anatomical elements; (2) the positions of the bones were broadly consistent with belonging to a single individual reclining on their back with the right upper limb crossed over the body (Fig. 3); (3) all elements derive from a skeletally mature (adult) individual; and (4) no teeth are duplicated in the assemblage and all anterior teeth are heavily worn, sharing an uncommon pattern of dental wear and pathological lesions. However, we acknowledge the possibility that the skeletal remains could pertain to multiple individuals. The seventh tooth, a molar, presents far less wear than the other teeth and lay below the scapula, and so likely came from a separate individual from Shanidar A.

#### The postcranial remains

**Fig. 4:**
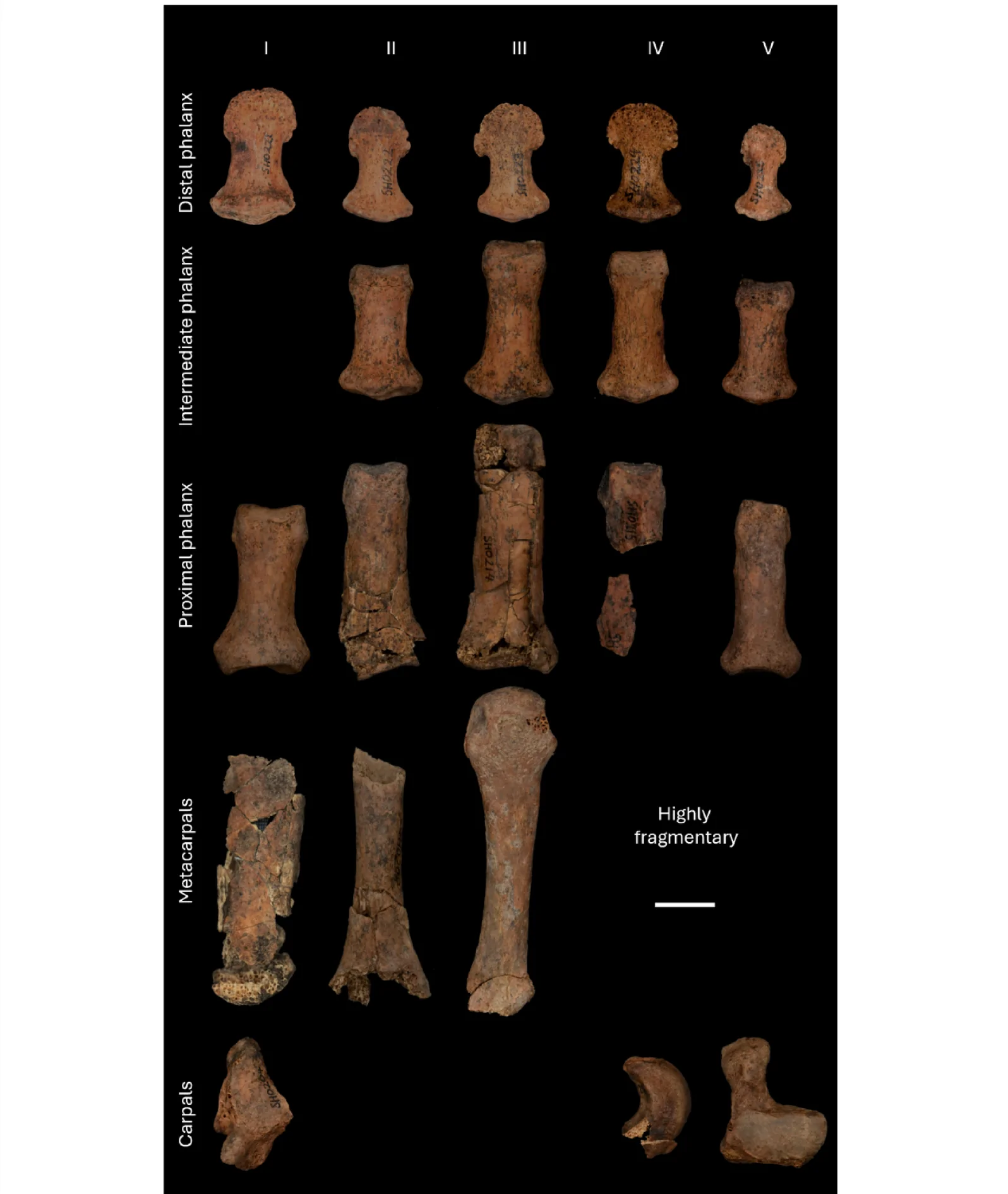
Right hand elements attributed to Shanidar A. Roman numerals denote digit number. Scale: 10 mm. Image: E. Pomeroy.

The right hand lay to the south of the left ribs with the hand flexed at the metacarpo-phalangeal joints. The phalanges lay on their dorsal sides, largely in anatomical position, but with some minor disturbance particularly affecting the first and fifth digits. The metacarpals lay upright with their dorsal surfaces against the large vertical rock to the south, and three preserved carpals lying above the level of the proximal metacarpal joint surfaces (trapezium, hamate and lunate) were scattered from their original anatomical positions (Fig. 3c). Photographs of the individual hand elements are given in Fig. 4.

**Fig. 5:**
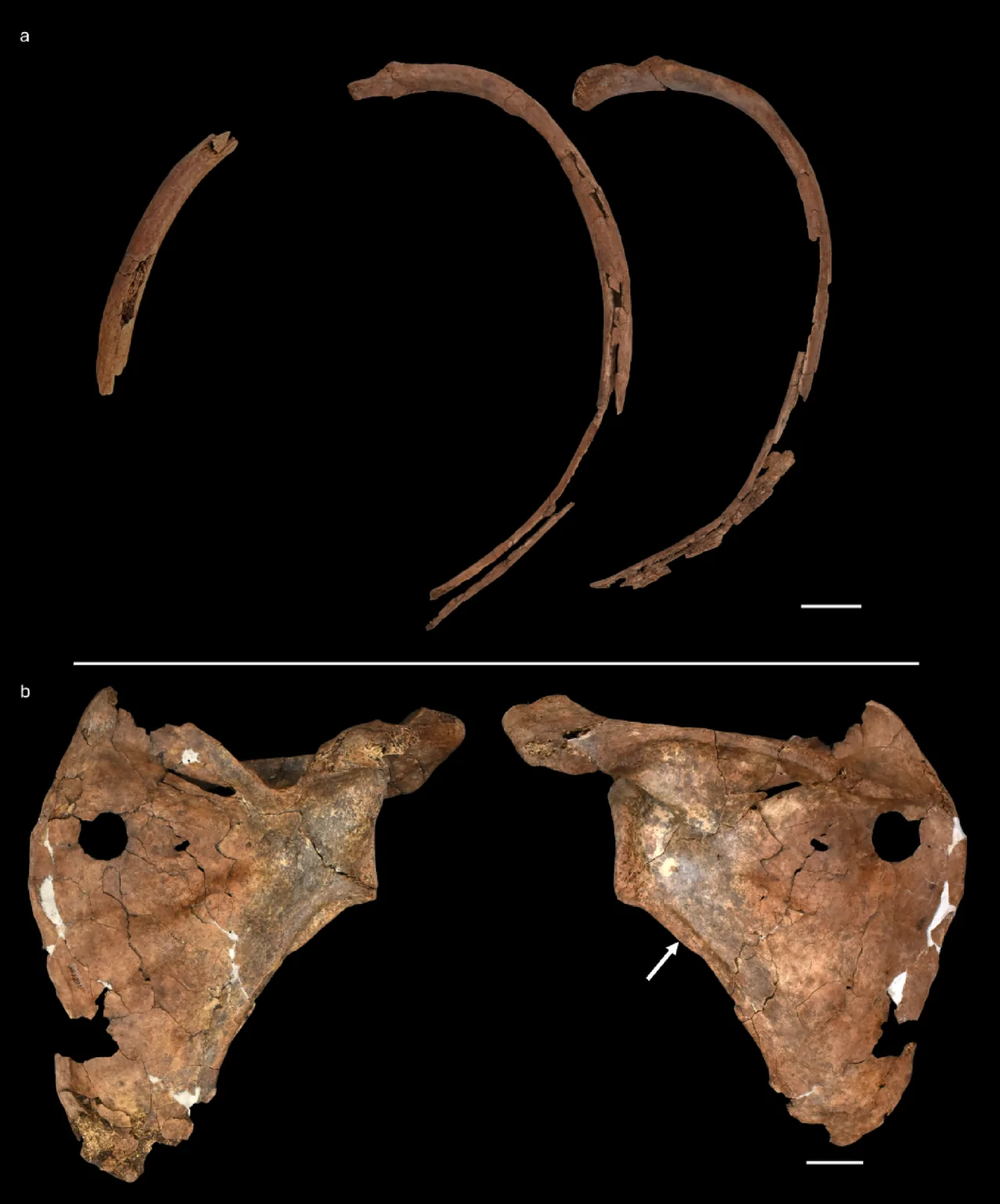
The ribs and scapula attributed to Shanidar A. **(a)** Cranial view of the fragment of right rib and the two left ribs. **(b)** Ventral (left) and dorsal (right) views of the scapula. The white arrow indicates the dorsal sulcus. White areas are where the scapula has been reconstructed with a calcium carbonate/Paraloid B72 mixture. Scales: 20 mm. Image: E. Pomeroy.

The left ribs are likely mid-thoracic (T6-8) based on their length, the narrow head and neck morphology of the more superior rib (superior both anatomically and as excavated), and the broadened head and neck morphology of the inferior rib (Fig. 5a). Both are highly fragmented and flattened but largely retain their overall morphology. The costal epiphyses are both fully fused indicating skeletal maturity, while the sternal ends are both missing. The position of the ribs relative to one another indicates that they were in anatomical position on discovery, and broadly consistent with a reclining body position. The right rib fragment comprises a section of shaft of a lower rib, beginning at the angle and extending sternally, that was displaced to the west (i.e., caudally) relative to the left ribs. The orientation of the long axis of the fragment aligns with the inferred direction of water flow, consistent with the fragment having been carried from its anatomical position by water (see below, ‘Sedimentary context and taphonomy’).

The scapula lay on its dorsal side slightly inclined to the east and in broad anatomical position relative to the two left ribs. The scapula is remarkably complete given its antiquity and the typical fragility of this element. It demonstrates a dorsal sulcus (Fig. 5b), a morphological trait that is common among Neanderthal individuals^21^, but not exclusive to them^22^.

**Fig. 6:**
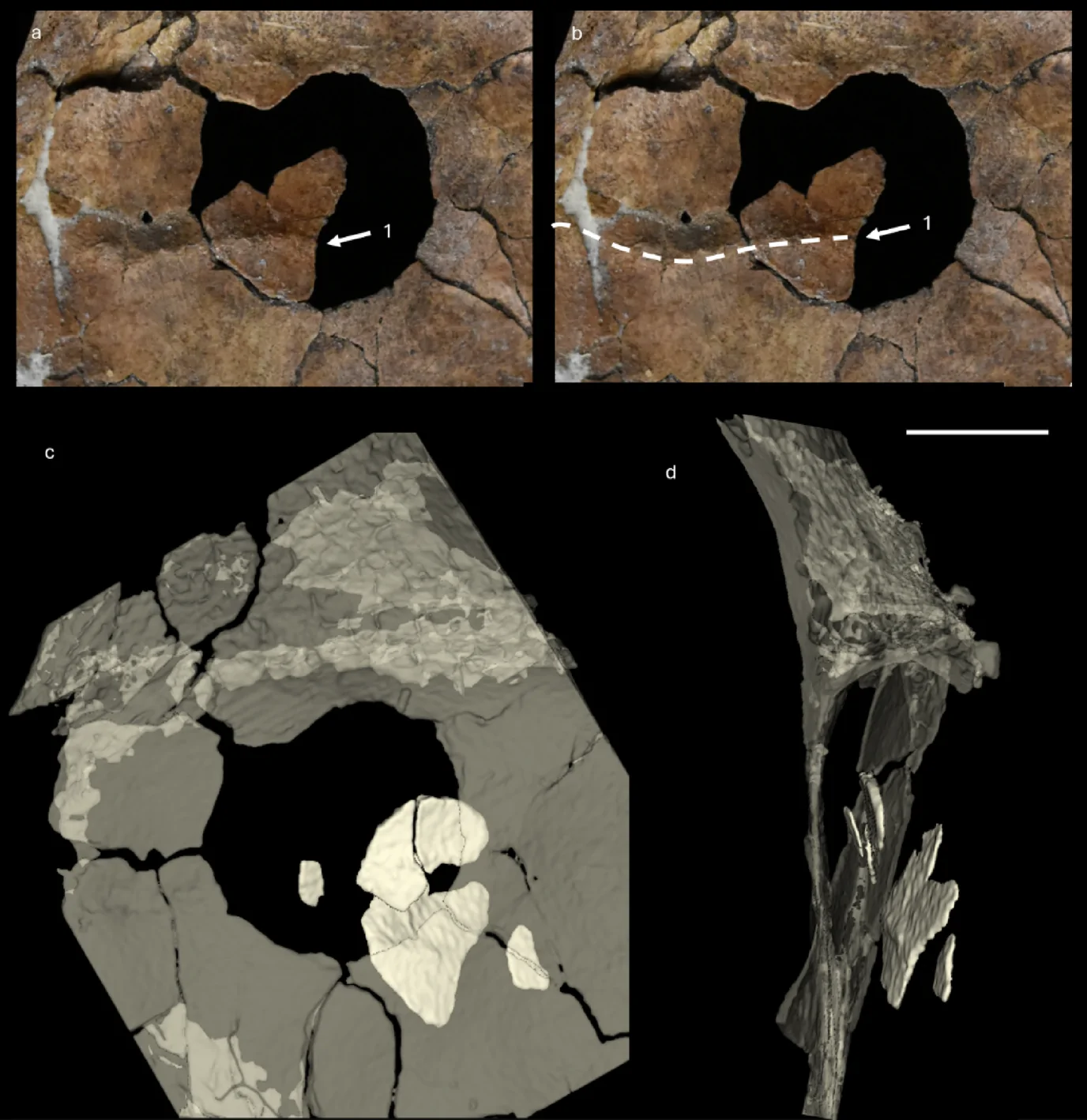
Refitting fragments embedded in the sediment below the perforation in the Shanidar A scapula. “1” indicates the same fragment in all images. **(a)** Ventral view of the perforation of the scapula, showing two refitting fragments. **(b)** The linea muscularis, highlighted by the dashed line, indicates that the fragments are correctly refitted. **(c)** 3D rendering of the perforation and fragments embedded in the sediment underneath the scapula *in situ* prior to conservation, in anterior view. The body of the scapula is semi-transparent to illustrate the positions of the fragments behind it. **(d)** 3D rendering showing the fragments originating from the perforation in the scapula in situ, viewed from the medial aspect. The dorsal side is to the right of the image. A video of the 3D rendering can be found in the Supplementary Information. Scale bar = 10 mm. Images: T. Van-Parys and E. Pomeroy.

The scapula, ribs and hand bones show no evidence of pathological lesions or degenerative processes. However, there is a perforation of the infraspinous fossa of the scapula measuring approximately 15 mm in diameter (Figs. 3b-c and 5) that displays a near-perfect circular outline, and from which at least three radial fractures extend. This hole was present at the time of discovery (Fig. 3b). The scapula was removed *en bloc* with underlying sediment, and micro-Computed Tomography (µCT) 3D reconstructions of the block showed the presence of at least four bone fragments, each measuring at least 4 mm in length, embedded in the sediment below the perforation. One of these fragments can confidently be refitted into the perforation (Fig. 6), confirmed by the presence of a *linea muscularis* on the ventral side of the scapula body that continues onto the fragment. Several other fragments recovered from beneath the scapula likely also came from the perforation, but further fragmentation of these small and fragile pieces during recovery and the conservation process means that their exact origin in the scapula cannot be discerned. The nature of this perforation is discussed further below (‘Analysis of the scapula perforation’).

## Dentition

**Fig. 7:**
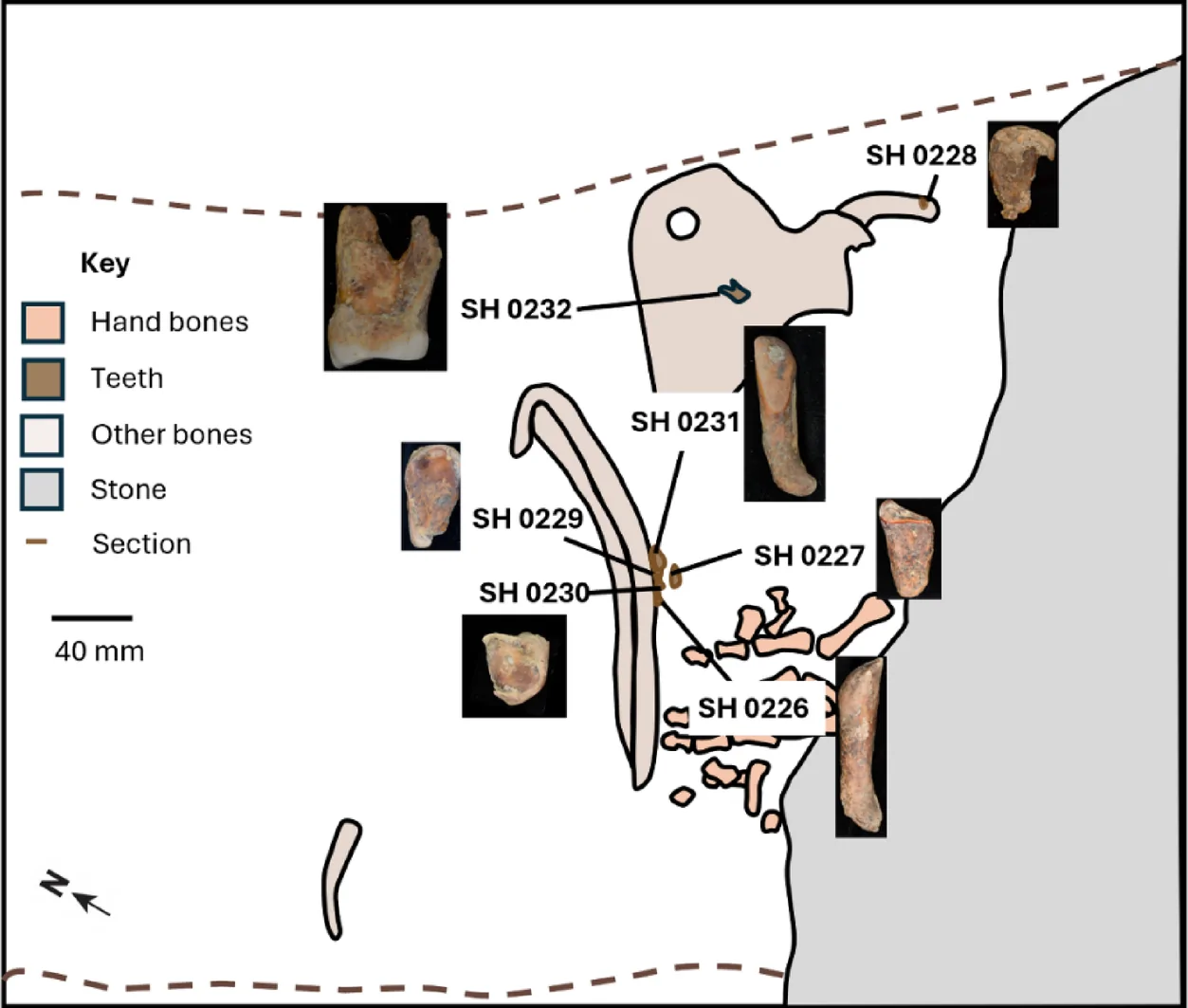
Enlargement of Fig. 3c showing the locations and inventory numbers of the teeth recovered (refer to Table S1 for details on individual teeth). Image: E. Pomeroy.

**Fig. 8:**
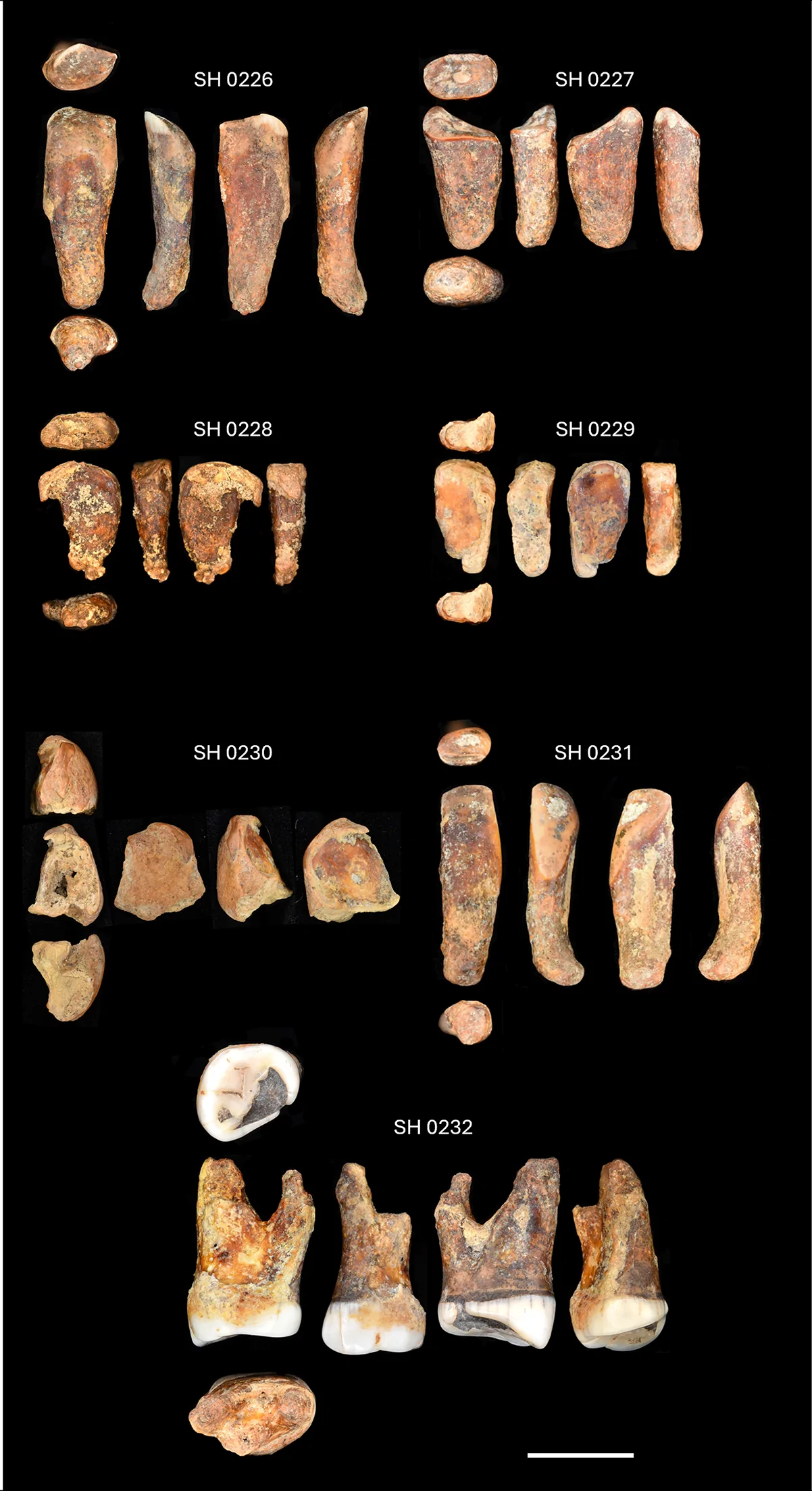
The dental remains attributed to Shanidar A, and associated molar recovered below the Shanidar A scapula. SH 0226: Mandibular right canine; SH 0227: Mandibular right lateral incisor; SH 0228: Mandibular right central incisor; SH 0229: Mandibular left central incisor; SH 0230: Mandibular left lateral incisor; SH 0231: Mandibular left canine; SH 0232: Maxillary left third molar. Views of each tooth are (from left to right): top: occlusal; bottom: apical; middle: mesial, labial, distal, lingual. Numbers prefixed with ‘SH’ are inventory numbers (see Table S1). Scale: 10 mm. Images: E. Pomeroy and N. Moss.

Six heavily worn, loose anterior teeth were found lying against the lateral aspect of the middle part of the rib shafts, and under the cranial part of the scapula. A third maxillary molar lay beneath the body of the scapula (Figs. 7 and 8). The molar has taurodont roots, a characteristic that is more common among Neanderthal individuals than in *Homo sapiens*^23^. The anterior teeth were extremely heavily worn in an unusual manner (Fig. 8, Table S2) even for Neanderthal teeth, which often display distinctive patterns of anterior dental attrition^24,25^.

The heavy wear on the anterior teeth is consistent with an older adult individual by Neanderthal standards (~ 45 years old), based on comparison with other Neanderthals from Shanidar Cave^14,25–27^. The lesser degree of wear on the maxillary third molar (SH 0232) suggests that this tooth came from another individual, as the disparity in wear between the anterior and posterior dentition is greater than seen in other Neanderthal individuals from Shanidar Cave (e.g., Shanidar 1, Shanidar 3). Sex could not be estimated from the elements recovered.

**Fig. 9:**
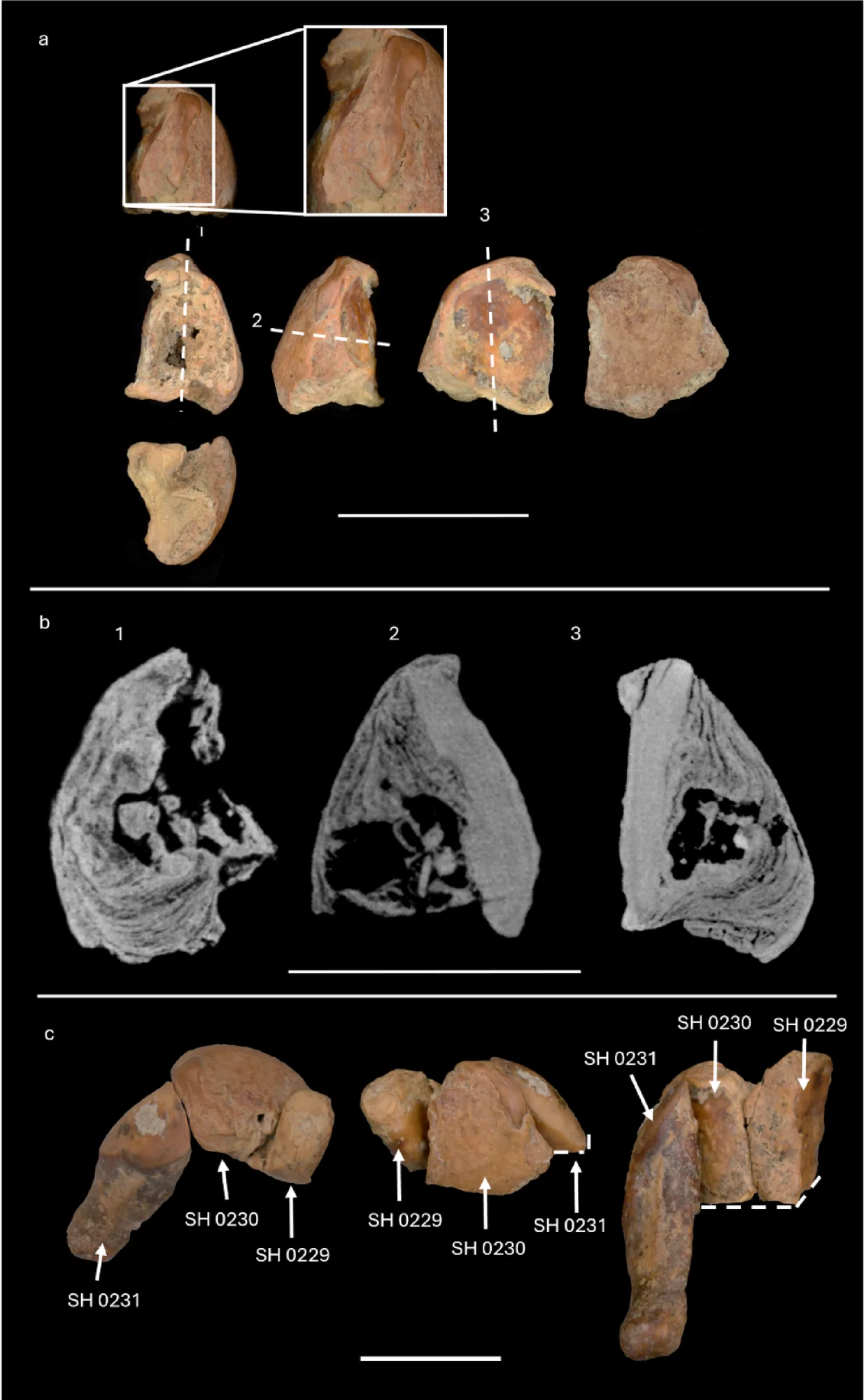
Shanidar A SH 0230 lateral incisor. (**a**) Photographs of the SH 0230 in (top to bottom and left to right) occlusal, mesial, apical, distal, lingual and labial views. Inset illustrates wear on the occlusal edge. (**b**) μCT tomographs of SH 0233 in the orientations illustrated in (a). (**c**) Reconstructed *in vivo* position of SH 0230 with SH 0229 and SH 0231 in (from left to right) occlusal, labial and lingual views. Dashed lines represent where teeth are truncated by material supporting them in position. Scales: 10 mm. Images: E. Pomeroy and N. Moss.

Some of the Shanidar A anterior teeth present heavy calculus, with one being almost entirely covered. This tooth, inventory number SH 0230, is half of a heavily worn incisor that was split longitudinally so that the mesial half was entirely lost. The mesial, labial, apical, and occlusal surfaces had become completely covered in calculus, indicating that the damaged tooth remained in the mouth for some time with all these surfaces exposed. The occlusal surface was still undergoing attritional wear resulting in polish on the occlusal surface, and which penetrated the thin calculus layer (Fig. 9), again, indicating the tooth had remained in the mouth and in use for some time following the trauma that damaged the tooth. The layered structure of the calculus is clearly visible in the µCT reconstructions (Fig. 9). The traumatic injury likely led to some rotation of this tooth and the adjacent left central incisor in the mouth and contributed to the unusual pattern of wear on the lower left canine, which lay tightly against the damaged incisor. Such damage to the tooth must have resulted from major trauma to the tooth and perhaps the front of the mandible, which likely contributed to the unusual anterior dental wear pattern. Although congenital crowding of the teeth may explain the position of the canine, such crowding is rare in Neanderthal and other hunter-gatherer dentitions (e.g.,^28^) and more usually associated with agriculturalist diets (e.g.,^29^). Therefore, the combination of severe damage to the lateral incisor alongside rotation of the incisors is most consistent with a traumatic injury.

In the absence of the accompanying mandibular and facial bones, and the maxillary and postcanine mandibular dentition, it is difficult to infer the nature of the trauma that likely resulted in the damage to the mandibular anterior dentition. Blunt force trauma, such as from an accident where the face or mouth was hit (e.g., a fall onto a hard surface) or from interpersonal violence, is a plausible explanation. Sharp force trauma cannot be excluded, although there is no evidence for this on the preserved teeth.

### Sedimentary context and taphonomy

Shanidar A, as with rest of the Shanidar 4/Z cluster, was found in the sediments infilling a shallow ephemeral alluvial channel inclined to the east. The relationships and positions of the bones of Shanidar A are consistent with being from a single individual. The predominantly preserved anatomical relationships among the skeletal elements suggest that on deposition, soft tissue was present. The bones of the hand, scapula and ribs were embedded in a fine-grained, weakly cross-laminated sand. This laminated sand is cut into by a much stonier unit that was deposited by an energetic and sediment-laden flash flood. This flood almost certainly flushed out most of the skeletal material, which by this point must have largely lacked soft tissue, and the position and orientation of the right rib fragment are consistent with this interpretation (cf.^30^). The bones of the hand were relatively protected by the vertical boulder and thus not disturbed when larger skeletal elements were removed by the current. The mandibular teeth were relocated by the current to come to rest against the ribs in a ‘log-jam’ (teeth are comparatively immobile in flowing water^31^), supporting our interpretation that the anterior teeth come from the same individual as the postcranial material.

The sedimentary and anatomical relationships of the bones upon excavation therefore suggest that the individual was deposited in the channel when the channel was dry, and that the body was incompletely covered with sediment from shallow wash very shortly after it was placed. Disturbance by the flash flood seems to have occurred after skeletonisation was largely complete and the sediments had dried out, since dry bones transport relatively easily in running water^32^. There was no evidence of a ‘grave cut’ to suggest intentional expansion of the channel to accommodate the body, in contrast with Shanidar Z^14^.

No evidence for carnivore activity (tooth marks, gnawing), nor anthropogenic surface modifications (including cut marks or percussion marks) was identified. The characteristic surface modifications and patterning of significant fluvial transport (sorting, abrasions, orientation^33^) were not identified other than in the orientation of the right rib fragment. Similarly, no traces of rodent activity or other biological agents, such as fungal or bacterial alteration, were observed.

Diagenetic modifications are largely restricted to mineral precipitation and post-depositional mechanical alteration. Manganese oxide precipitation is widespread across the assemblage, and calcium carbonate precipitation was recorded below the scapula and on one rib and one incisor. There is no evidence of dissolution, weathering, trampling, or abrasion of bone surfaces by sedimentary particles. Several elements are crushed or flattened (Figs. 4 and 5), consistent with compression under sedimentary load, while remaining largely complete. Other elements are incomplete and display fractures that, although not strongly diagnostic, are generally consistent with post-mortem breakage, based on the irregular morphology of the fracture surfaces and the absence of characteristics associated with fresh bone fracture.

The absence of evidence of scavenger or rodent action affecting any of the Neanderthal skeletal remains in the mortuary cluster (Shanidar 4, 6, 8, 9^25^, A, B and Z) and any of the broadly contemporaneous faunal remains indicates that animal involvement is an unlikely explanation for the spatial positioning of the hominin bones. The absence of evidence for significant water disturbance of the surviving skeletal material is unsurprising given that we interpret the skeletal elements that we recovered as being largely *in situ* since they retain their relative anatomical positions, aside from minor movements of the hand elements and right rib. The scattered positions of the anterior teeth, lying upstream to, but caught against, the skeletal elements (Fig. 3c), are consistent with the scattering of the teeth and their movement by water until they accumulated against an obstruction (cf.^30^). The lack of water rolling damage to the teeth is consistent with their being carried over a short distance by a burst of fast-moving water. Fluvial deposition as an explanation for the positions of most of the skeletal remains also seems unlikely, especially given the lack of shared orientation that would be expected if the bones were carried by a water current^30,33^, and the remarkable preservation of the scapula. The exception is the position of the right rib, which is entirely consistent with having been disturbed from its anatomical position by such a water current.

The finding of displaced, individual teeth is common at Neanderthal and other Palaeolithic sites^34–36^, as presumably once they came loose from the skull their small size meant that they would have been liable to be transported by water or other means, while their robust nature ensured that they preserve while the maxillary or mandibular bones do not. The displacement of the right carpals, as well as rays 1 and 5 of the hand, again indicates post-depositional disturbance, potentially by the same water flow, but at the same time the remains were partially covered by sediment that protected most of the metacarpals and phalanges from such disturbance.

### Analysis of the scapula perforation

**Fig. 10:**
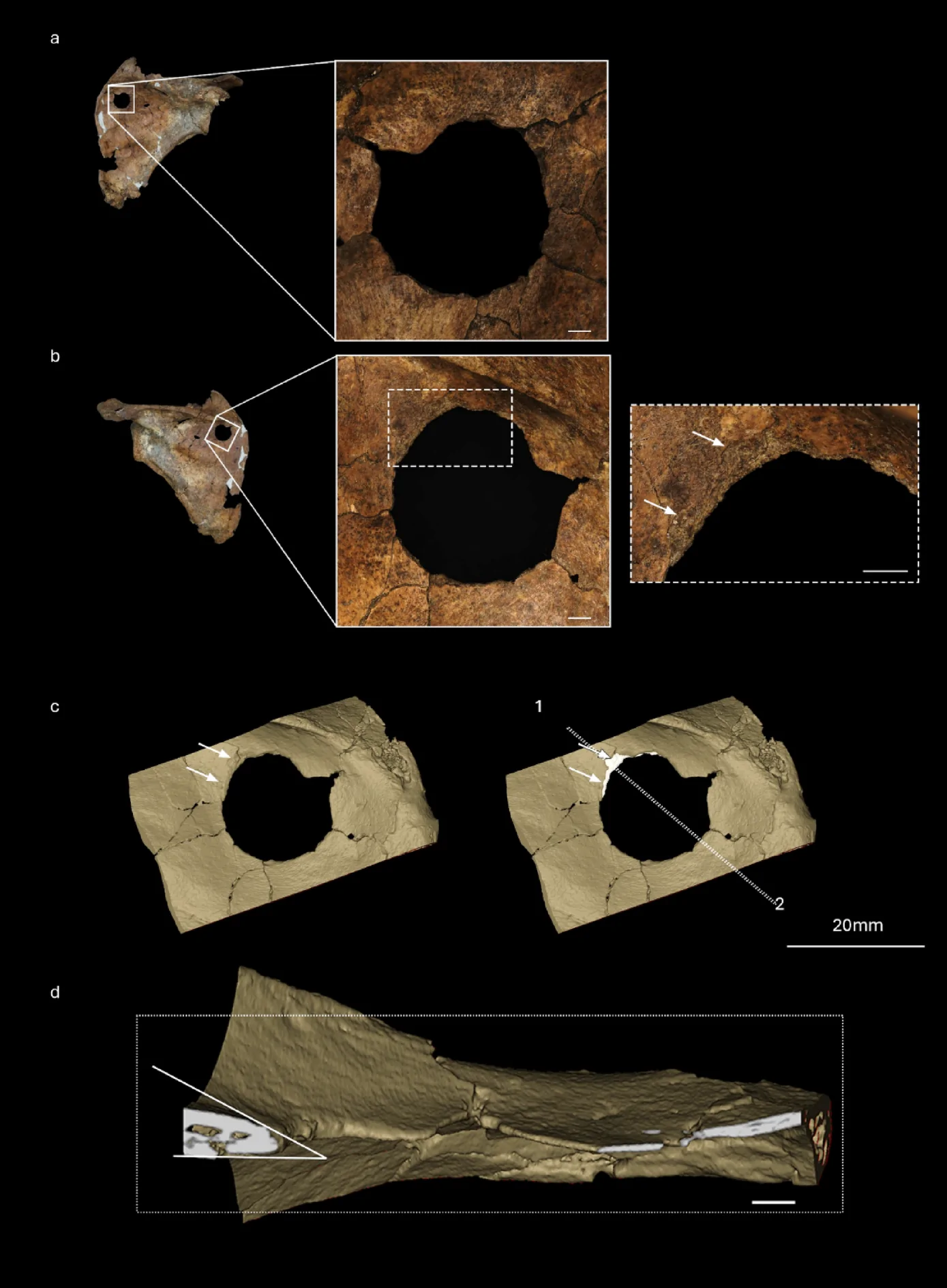
Detail of the hole observed on the left scapula of Shanidar A. **(a)** Ventral view and **(b)** dorsal view of the scapula, showing the perforation. White arrows indicate the edge of the bevelled area. **(c)** µCT image of the perforation, with the bevelled margin indicated by white arrows; the area affected by bevelling is highlighted in white, and numbers 1 and 2 mark the location of the µCT section shown in **(d)**. **(d)** µCT section showing part of the perforation edge affected by bevelling, where an acute fracture angle can be observed. Scale bars are 2 mm unless otherwise indicated. Image: A. Pantoja-Pérez and N. Sala.

There was no evidence of recent damage to the perforated area of the scapula, nor of, for example, insect burrows or plant roots of appropriate diameter penetrating the sediment in the location of the hole that would suggest that the perforation resulted from burrowing or plant growth. The absence of burrows or plant roots of a similar diameter to the perforation passing through the hole was confirmed by µCT reconstructions of the underlying sediment. Macroscopic, microscopic and µCT analyses of the scapula allowed detailed characterisation of the perforation and associated fractures. The margins of the perforation are smooth and show mineral precipitation, including manganese oxides, consistent with the diagenetic alterations observed across the rest of the bone surface, indicating that the perforation was most likely formed prior to burial, or at least is not recent in origin. Microscopic and µCT examination of the perforation revealed no evidence of bone remodelling, periosteal reaction, or irregular woven bone associated with the margins of the perforation which would indicate healing (Fig. 10a-b), meaning that the perforation occurred either peri- or postmortem.

The perforation is best explained as resulting from perimortem penetrating trauma, such as that caused by a projectile or a thrusting implement such as a spear. The bevelling on the posterior side of the perforation (Fig. 10) indicates a ventral-to-dorsal trajectory of the implement^37^occurring perimortem, since bevelling is typical of the exit wound in cases of this kind of trauma (e.g.,^38^), while the radiating fractures are also typical of perimortem trauma^39^.

The fragments of bone originating from the perforation and situated directly below it in the sediment are consistent with having been displaced by a puncture wound and becoming lodged in the soft tissue covering the scapula. The fragments were displaced slightly laterally and caudally from the perforation and angled away from the centre of the hole. This displacement suggests that the weapon causing the wound entered the body from a medial and superior angle relative to Shanidar A’s body. If the weapon was pulled out of the body from the front, this could have displaced the fragments further. The fragments are then likely to have been only minimally disturbed during the decay process. The fine sandy sediment in which the body lay would likely have trickled in to fill the space created as the tissues gradually decayed^40,55^. Although the fragments might have been relocated by the moving water that displaced the teeth and other parts of the skeleton, the fact that the scapula is likely in anatomical position and orientation relative to the ribs suggests that the small, light and easily transported fragments are likely to be in the positions in which they arrived immediately after Shanidar A received the wound, protected from fluvial disturbance by the scapular spine which lay upstream of the fragments.

Very similar lesions have been documented in the remains of large, medium and small fauna from several Palaeolithic and Mesolithic sites, especially in the scapula^41–43^, and recreated experimentally using wooden, bone- and lithic-tipped projectiles and thrusting spears^38,44–48^. Published archaeological lesions are generally circular, oval or lozenge-shaped, with radiating fractures, bevelling on the side of the likely exit wound, and even in some cases a notched appearance on one side of the perforation reminiscent of the lesion in Shanidar A^41–43,49^. Noe-Nygaard^41^ speculates that the circular and notched lesion on a red deer (*Cervus elaphus*) scapula may result from a barbed projectile, though supporting evidence for this interpretation is lacking. Perforations are also fairly common on scapulae and have been interpreted as resulting from a ‘heart shot’ aimed at incapacitating prey and causing their swift death^41,49^.

Based on experimental evidence^38,48^, the circular shape of the perforation observed on Shanidar A’s scapula is consistent with a wound from a wooden spear of rounded cross-sectional shape, rather than by a stone-tipped weapon with the typically ovate or lozenge shape and potentially narrow-edged profile in cross-section of a hafted lithic^46,48,50^. Bone tipped spears are less well documented from the Middle Palaeolithic, but based on the example from Abric Romaní^51^, would have a cross-section more similar to lithic points. Wooden spears with rounded cross sections, potentially shaped for both thrusting and throwing, are documented in Europe as early as 400 ka at Clacton-on-Sea, UK^52,53^ and 200 ka in the case of the spears and other wooden artefacts from Schöningen, Germany^54–56^. It is notable that a narrow-edged lesion observed in the Shanidar 3 individual’s left 9th rib is interpreted as an injury caused by a lithic-tipped projectile^25,57^.

## Discussion

As Shanidar 4, Z, A and B are all skeletally mature and all preserve a common section of the acromion process of the left scapula, while Shanidar 9 is a young infant^25^, the discovery of Shanidar A brings the minimum number of individuals in the cluster to five. Whether Shanidar 6 and 8 represent partial remains of some of the same individuals is unclear, and detailed study of the 1960 remains, now curated in the Iraq Museum in Baghdad, is planned to investigate whether the Shanidar Z, A and B remains could belong to the individuals originally designated Shanidar 6 and 8. However, a lack of information regarding the original positions of Shanidar 6, 8 and 9 upon excavation and likely damage to potentially refitting surfaces when the Shanidar 4, 6, 8 and 9 remains were removed in 1960 will make this task very challenging^16^.

The Shanidar A remains represent the earliest in the depositional sequence within the cluster. These remains attributed to Shanidar A are consistent with belonging to a single individual (likely with the exception of the third molar) whose body decomposed *in situ*, at least in part, given the retained anatomical position among the skeletal elements and the sedimentary context. The incompleteness of the remains and the positions of the teeth are consistent with the skeleton having been disturbed by a flash flood. The extremely heavy attrition of the anterior teeth suggests an older individual, like the overlying Shanidar Z individual.

The pattern of dental trauma and subsequent calculus accumulation points to very unusual pathology following a major facial injury, which the individual survived, while the perforation to the scapula indicates traumatic injury around the time of death and likely causing it. In general, Neanderthal individuals and Middle-Upper Palaeolithic modern humans show a high frequency of traumatic injury, but these injuries are mostly linked to probable accidents, perhaps due to hunting at close quarters^58,59^. Whether the traumatic lesion on the scapula resulted from something like a hunting accident or from interpersonal violence cannot be known. Either way, the spear would have entered the individual’s body on the left-hand side whilst they were facing forward. Such an injury would have penetrated all the way through the chest, including the lungs and potentially major blood vessels, likely leading to a rapid death. To develop more robust interpretations regarding the mechanism responsible for this perforation, ongoing experimental work in forensic taphonomy is exploring whether the observed morphological features could be produced under high kinetic energy conditions, as opposed to low kinetic energy impacts, which will help to constrain the range of processes capable of generating similar perforations in human bone.

This is the second case of perforating trauma from Shanidar Cave alongside the puncture wound to Shanidar 3’s ribcage^25,57,60^, although it should be noted that the Shanidar 3 remains are around 25 ka younger than those of Shanidar A. While there has been speculation that Shanidar 3’s puncture wound could be the result of violent interactions between Neanderthals and anatomically modern humans, in reality, violence between individuals and/or between groups, including with other Neanderthals, cannot be demonstrated. The perforation to the Shanidar A scapula adds to a very small number of examples of possible interpersonal violence in the Middle-Late Pleistocene, including the cranial trauma among the Sima de los Huesos pre-Neanderthals^61,62^, the Saint Césaire Neanderthal^63^, the Qafzeh 25 Middle Palaeolithic anatomically modern human^64^, and Upper Palaeolithic modern humans^65–70^, although not all potential cases are straightforward to identify^71,72^. The presence of possible interpersonal trauma feeds into debates concerning the origins of both interpersonal violence and care^57,73^ in the hominin lineage. Shanidar A’s shoulder injury appears to have been fatal, but the apparent placement of the body in a location that subsequently became part of a tight cluster of Neanderthal individuals, who were likely intentionally deposited within living memory of each other, strongly suggests a level of care during and immediately after death. The facial injury that severely damaged the anterior mandibular dentition may have also required a level of care during the period of recovery and potentially afterwards, given the extent of the damage to the teeth and the impact this may have had on communication abilities, eating and pain levels long-term.

Finally, the potential use of a wooden projectile or thrusting spear provides indirect evidence of the use of organic weapons at Shanidar Cave 75 ka, which was likely in the light of the Schöningen and Clacton-on-Sea spears but extremely difficult to demonstrate at many Middle Palaeolithic sites given a lack of significant organic preservation here beyond pollen and charred wood. In addition to Clacton-on-Sea and Schöningen, wooden ‘throwing sticks’ dating to c. 130 ka were found at Poggetti Vecchi, Italy^74^ and a digging stick dated to 130 − 50 ka at Aranbaltza III, Spain (51 − 42 ka)^75^. Other evidence for Middle Palaeolithic use of organic objects includes pseudomorphs of wooden objects with uncertain purpose from Abric Romaní, dated to around 47–49 ka^76^, while Abri du Maras, France, provides currently unique evidence of the use of twine^77^. Lithic use wear analyses provide evidence of Middle Palaeolithic woodworking^78^, although such evidence provides little indication of what was being produced.

The traumatic injuries, both non-fatal and fatal, the accumulating evidence for possibly symbolic mortuary behaviour, and the indirect evidence for the use of wooden weapons 75 ka, provide significant new perspectives on the nature of Neanderthal life and death at Shanidar Cave.

## Methods

### Excavation and conservation of the Neanderthal remains

Excavations were undertaken using the single context method^79^. The bones were extremely soft and fragile, so were consolidated *in situ* using an approximately 20% solution of Paraloid B72 in acetone, with the addition of cotton gauze to stabilise the scapula. The elements were then removed in small blocks with the surrounding sediment (scapula and left ribs) or individually (right rib, teeth that could be easily removed, hand bones). The blocks, hand bones and teeth were imaged using µCT prior to and after cleaning and conservation^80^ in the Palaeoanthropology Laboratory, Department of Archaeology, University of Cambridge using imaging facilities in the Palaeoanthropology Laboratory (Bruker Skyscan 1273) and at the Cambridge Biotomography Centre, University of Cambridge (Nikon XTEK 225 ST). The removal of the scapula remains with the underlying sediment and subsequent µCT imaging allowed for the visualisation of the fragments underlying the perforation.

The µCT 3D reconstruction was visualised in Thermo Scientific Avizo Software [2022.2] and 3D Slicer v. 4.1.0 (slicer.org). The segmentation of the scapula perforation and underlying fragments was performed in 3D Slicer using the SlicerNNIneractive extension^81,82^. The video SI2 was created in 3D Slicer.

### Dental wear

Dental wear was assessed following Molnar^83^.

### Taphonomic alterations

The analysis of bone surface modifications followed established taphonomic standards and methodological criteria widely applied in palaeoanthropological contexts (^84^ and references therein), and employed macroscopic and microscopic examination with a binocular stereomicroscope (Nikon SMZ745T). Fracture pattern analysis followed the criteria proposed by Bunn^85^ and Villa and Mahieu^86^ as applied in recent taphonomic studies of hominin assemblages^62,84,87,88^, including the assessment of fracture outline, angle, and edge morphology. Further details are given in the Supplementary Methods.

Data supporting the findings of this study are available within the article or Supplementary Information.

## Supplementary Information

Below is the link to the electronic supplementary material.

Supplementary Material 1

Supplementary Material 2

Supplementary Material 3

Supplementary Material 4

## Data Availability

All data supporting the findings of this study are available within the paper and its Supplementary Information.

## References

[1] Pettitt, P. *The Palaeolithic Origins of Human Burial* (Routledge, 2011).

[2] Zilhão, J. Lower and Middle Palaeolithic mortuary behaviours and the origins of ritual burial in Death Rituals, Social Order and the Archaeology of Immortality in the Ancient World: ‘Death Shall Have No Dominion (ed. Renfrew, C., Morley, I., & Boyd, M.J.) 27–44Cambridge University Press, (2015).

[3] Dirks, P. H. G. M. et al. Geological and taphonomic context for the new hominin species *Homo naledi *from the Dinaledi Chamber, South Africa. *eLife* **4**, e09561. (2015). 10.7554/eLife.09561 PMC4559842 26354289

[4] Pettitt, P. Did *Homo naledi* dispose of their dead in the Rising Star Cave system? *Afr. J. Sci.* **118**, 1–3 (2022).

[5] Roebroeks, W. & Soressi, M. Neandertals revised. *Proc. Natl. Acad. Sci. U S A*. **113**, 6372–6379 (2016). 10.1073/pnas.1521269113 PMC4988603 27274044

[6] Pomeroy, E. et al. Issues of theory and method in the analysis of Paleolithic mortuary behavior: A view from Shanidar Cave. *Evol. Anthropol.* **29**, 263–279 (2020). 10.1002/evan.21854 32652819

[7] Solecki, R. S. & Shanidar *The First Flower People* (Knopf, Inc., 1971).

[8] Gargett, R. H. Grave shortcomings: The evidence for Neandertal burial. *Curr. Anthropol.* **30**, 157–190 (1989).

[9] Gargett, R. H. Middle Palaeolithic burial is not a dead issue: The view from Qafzeh, Saint-Césaire, Kebara, Amud, and Dederiyeh. *J. Hum. Evol.* **37**, 27–90 (1999). 10.1006/jhev.1999.0301 10375476

[10] Hovers, E., Kimbel, W. H. & Rak, Y. The Amud 7 skeleton - still a burial. J Hum. Evol39, 254–260 (2000) 10.1006/jhev.1999.0406 10968931

[11] Dibble, H. L. et al. A critical look at evidence from La Chapelle-aux-Saints supporting an intentional Neandertal burial. *J. Archaeol. Sci.* **53**, 649–657 (2015).

[12] Goldberg, P. et al. Testing the Roc de Marsal Neandertal burial with geoarchaeology. *Archaeol. Anthropol. Sci.* **9**, 1005–1015 (2017).

[13] Chase, P. G. & Dibble, H. L. Middle Paleolithic symbolism: A review of current evidence and interpretations. *J. Anthropol. Archaeol.* **6**, 263–296 (1987).

[14] Pomeroy, E. et al. New Neanderthal remains associated with the ‘flower burial’ at Shanidar Cave. *Antiquity* **94**, 11–26 (2020).

[15] Barker, G. et al. Neanderthal life and death in Shanidar Cave, Iraqi Kurdistan [abstract]. *PaleoAnthropology, ***2023,** 283. (2023).

[16] Pomeroy, E. et al. Excavating the archives: New perspectives on the Shanidar Cave Neanderthals. Bull. Mém Soc. Anthropol. Paris. 37, 1 (2025)

[17] Sommer, J. D. The Shanidar IV ‘flower burial’: A re-evaluation of Neanderthal burial ritual. *Camb. Archaeol. J.* **9**, 127–129 (1999).

[18] Solecki, R. S. & Shanidar, I. V. a Neanderthal flower burial in northern Iraq. *Science* **190**, 880–881 (1975).

[19] Leroi-Gourhan, A. The flowers found with Shanidar IV, a Neanderthal burial in Iraq. *Science* **190**, 562–564 (1975).

[20] Hunt, C. O., Pomeroy, E., Reynolds, T., Tilby, E. & Barker, G. Shanidar et ses fleurs? Reflections on the palynology of the Neanderthal ‘Flower Burial’ hypothesis. *J. Archaeol. Sci.* **159**, 105822 (2023).

[21] Trinkaus, E. A functional interpretation of the axillary border of the Neandertal scapula. *J. Hum. Evol.* **6**, 231–234 (1977).

[22] Moran, A. J. & Chamberlain, A. T. The incidence of dorsal sulci of the scapula in a modern human population from Ensay, Scotland. *J. Hum. Evol.* **33**, 521–524 (1997). 10.1006/jhev.1997.0175 9361259

[23] Kupczik, K. & Hublin, J. J. Mandibular molar root morphology in Neanderthals and Late Pleistocene and recent *Homo sapiens*. *J. Hum. Evol.* **59**, 525–541 (2010). 10.1016/j.jhevol.2010.05.009 20719359

[24] Ungar, P. S., Fennell, K. J., Gordon, K. & Trinkaus, E. Neandertal incisor beveling. *J. Hum. Evol.* **32**, 407–421 (1997). 10.1006/jhev.1996.0109 9169991

[25] Trinkaus, E. *The Shanidar Neandertals* (Academic, 1983).

[26] Trinkaus, E. & Thompson, D. D. Femoral diaphyseal histomorphometric age determinations for the Shanidar 3, 4, 5, and 6 Neandertals and Neandertal longevity. *Am. J. Phys. Anthropol.* **72**, 123–129 (1987). 10.1002/ajpa.1330720115 3103459

[27] Thompson, D. & Trinkaus, E. Age determination of the Shanidar 3 Neanderthal. *Science* **212**, 575–577 (1981). 10.1126/science.6782677 6782677

[28] Frayer, D. W., Gatti, J., Monge, J., Radovčić, D. & Prehistoric dentistry? P4 rotation, partial M3 impaction, toothpick grooves and other signs of manipulation in Krapina Dental Person 20. *Bull. Int. assoc. paleodont*. **11**, 1–10 (2017).

[29] von Cramon-Taubadel, N. Global human mandibular variation reflects differences in agricultural and hunter-gatherer subsistence strategies. *Proc. Natl. Acad. Sci. U S A*. **108**, 19546–19551 (2011). 10.1073/pnas.1113050108 PMC3241821 22106280

[30] Chiappone, M., Guala, M., Rogers, R. & Makovicky, P. When the levee breaks: experimentally testing dinosaur and mammal bone transport in unsteady flows. *Paleobiology* **52**, 299–314 (2026).

[31] Behrensmeyer, A. K. Taphonomy and paleoecology of Plio-Pleistocene bone assemblages of Lake Rudolph, Kenya. *Bull. Mus. Comp. Zool.* **146**, 473–578 (1975).

[32] Coard, R. One bone, two bones, wet bones, dry bones: Transport potentials under experimental conditions. *J. Archaeol. Sci.* **26**, 1369–1375 (1999).

[33] Behrensmeyer, A. K. Vertebrate preservation in fluvial channels. *Palaeogeogr Palaeoclimatol Palaeoecol*. **63**, 183–199 (1988).

[34] Henrion, J. et al. The Grotte du Bison Neandertals (Arcy-sur-Cure, France). *J. Hum. Evol.* **199**, 103631 (2025). 10.1016/j.jhevol.2024.103631 39729880

[35] Zanolli, C. et al. A Neanderthal from the Central Western Zagros, Iran. Structural reassessment of the Wezmeh 1 maxillary premolar. *J. Hum. Evol.* **135**, 102643 (2019). 10.1016/j.jhevol.2019.102643 31421316

[36] Garralda, M. D. et al. The Neanderthal teeth from Marillac (Charente, Southwestern France): Morphology, comparisons and paleobiology. *J. Hum. Evol.* **138**, 102683 (2020). 10.1016/j.jhevol.2019.102683 31765984

[37] Christensen, A. M. & Rickman, J. M. Cone cracking in human bone: A CT case review series. *Foren Imag.* **30**, 200510 (2022).

[38] Gaudzinski-Windheuser, S. et al. Evidence for close-range hunting by last interglacial Neanderthals. *Nat. Ecol. Evol.* **2**, 1087–1092 (2018). 10.1038/s41559-018-0596-1 29942012

[39] Sorg, M. H. Differentiating trauma from taphonomic alterations. *Forensic Sci. Int.* **302**, 109893 (2019). 10.1016/j.forsciint.2019.109893 31419593

[40] Duday, H. *The Archaeology of the Dead: Lectures in Archaeothanatology. Translated by Anna Maria Cipriani and John Pearce* (Oxbow Books, 2009).

[41] Noe-Nygaard, N. Mesolithic hunting in Denmark illustrated by bone injuries caused by human weapons. *J. Archaeol. Sci.* **1**, 217–248 (1974).

[42] Noe-Nygaard, N. Two shoulder blades with healed lesions from Star Carr. *Proc. Prehist Soc.* **41**, 10–16 (1975).

[43] Gaudzinski-Windheuser, S. in *in Multidisciplinary Approaches to the Study of Stone Age Weaponry*. 77–100 (eds Iovita, R. & Sano, K.) (Springer Netherlands, 2016).

[44] Letourneux, C. & Pétillon, J. M. Hunting lesions caused by osseous projectile points: experimental results and archaeological implications. *J. Arch. Sci.* **35**, 2849–2862 (2008).

[45] Smith, G. Damage inflicted upon animal bone by wooden projectiles: Experimental results and archaeological applications. *J. Taphon.* **1**, 3 –12 (2003).

[46] Smith, G. M. et al. When lithics hit bones: Evaluating the potential of a multifaceted experimental protocol to illuminate Middle Palaeolithic weapon technology. *J. Paleolit Archaeol.* **3**, 126–156 (2020).

[47] Yeshurun, R. et al. Identification and quantification of projectile impact marks on bone: new experimental insights using osseous points. *Archaeol. Anthropol. Sci.* **16** 10.1007/s12520-024-01944-3 (2024). PMC10884158 38404950

[48] Duches, R. et al. Identification of Late Epigravettian hunting injuries: Descriptive and 3D analysis of experimental projectile impact marks on bone. *J. Archaeol. Sci.* **66**, 88–102 (2016).

[49] Leduc, C. New Mesolithic hunting evidence from bone injuries at Danish Maglemosian sites: Lundby Mose and Mullerup (Sjælland). *Int. J. Osteoarchaeol*. **24**, 476–491 (2014).

[50] Smith, M. J., Brickley, M. B. & Leach, S. L. Experimental evidence for lithic projectile injuries: Improving identification of an under-recognised phenomenon. *J. Archaeol. Sci.* **34**, 540–553 (2007).

[51] Mateo-Lomba, P. et al. First identification of a Neanderthal bone spear point through an interdisciplinary analysis at Abric Romaní (NE Iberian Peninsula). *Sci. Rep.* **14**, 19160 (2024). 10.1038/s41598-024-67817-w PMC11333748 39160167

[52] Bridgland, D. R. et al. Middle Pleistocene interglacial Thames–Medway deposits at Clacton-on-Sea, England: Reconsideration of the biostratigraphical and environmental context of the type Clactonian Palaeolithic industry. *Quat Sci. Rev.* **18**, 109–146 (1999).

[53] Allington-Jones, L. The Clacton spear: The last one hundred years. *Archaeol. J.* **172**, 273–296 (2015).

[54] Hutson, J. M. et al. Revised age for Schöningen hunting spears indicates intensification of Neanderthal cooperative behavior around 200,000 years ago. *Sci. Adv.* **11**, eadv0752 (2025). 10.1126/sciadv.adv0752 PMC12063642 40344053

[55] Thieme, H. Lower Palaeolithic hunting spears from Germany. *Nature* **385**, 807–810 (1997). 10.1038/385807a0 9039910

[56] Leder, D. et al. The wooden artifacts from Schöningen’s Spear Horizon and their place in human evolution. *Proc. Natl. Acad. Sci. U S A*. **121**, e2320484121 (2024). 10.1073/pnas.2320484121 PMC11009636 38557183

[57] Churchill, S. E., Franciscus, R. G., McKean-Peraza, H. A., Daniel, J. A. & Warren, B. R. Shanidar 3 Neandertal rib puncture wound and paleolithic weaponry. *J. Hum. Evol.* **57**, 163–178 (2009). 10.1016/j.jhevol.2009.05.010 19615713

[58] Beier, J., Anthes, N., Wahl, J. & Harvati, K. Similar cranial trauma prevalence among Neanderthals and Upper Palaeolithic modern humans. *Nature* **563**, 686–690 (2018). 10.1038/s41586-018-0696-8 30429606

[59] Trinkaus, E. & Neandertals early modern humans, and rodeo riders. *J. Archaeol. Sci.* **39**, 3691–3693 (2012).

[60] Stewart, T. D. Fossil evidence of human violence. *Trans-action* **6**, 48–53 (1969).

[61] Sala, N. et al. Lethal interpersonal violence in the Middle Pleistocene. *PLOS ONE*. **10**, e0126589 (2015). 10.1371/journal.pone.0126589 PMC4446311 26018668

[62] Sala, N., Pantoja-Pérez, A., Gracia, A. & Arsuaga, J. L. Taphonomic-forensic analysis of the hominin skulls from the Sima de los Huesos. *Anat. Rec*. **307**, 2259–2277 (2024). 10.1002/ar.24883 35195943

[63] Zollikofer, C. P. E., Ponce de León, M. S., Vandermeersch, B. & Lévêque, F. Evidence for interpersonal violence in the St. Césaire Neanderthal. *Proc. Natl. Acad. Sci. U S A*. **99**, 6444–6448 (2002). 10.1073/pnas.082111899 PMC122968 11972028

[64] Pantoja-Pérez, A., Martín-Francés, L., May, H., Hershkovitz, I. & Sala N. A taphonomic reassessment of Qafzeh 25 and its implications for violence, health and funerary practices. *Sci. Rep.* **16**, 13908 (2026). 10.1038/s41598-026-58670-0 PMC13623648 42380181

[65] Trinkaus, E. & Buzhilova, A. P. The death and burial of Sunghir 1. *Int. J. Osteoarchaeol*. **22**, 655–666 (2012).

[66] Kranioti, E. F., Grigorescu, D. & Harvati, K. State of the art forensic techniques reveal evidence of interpersonal violence ca. 30,000 years ago. *PLOS ONE*. **14**, e0216718 (2019). 10.1371/journal.pone.0216718 PMC6608943 31269019

[67] Knüsel, C. J., Thibeault, A. & Villotte, S. A cranial injury from the earliest Gravettian at the Cro-Magnon rock shelter (Vézère Valley, Dordogne, southwest France). *J. Hum. Evol.* **177**, 103329 (2023). 10.1016/j.jhevol.2023.103329 36870149

[68] Sparacello, V. S. et al. Projectile weapon injuries in the Riparo Tagliente burial (Veneto, Italy) provide early evidence of Late Upper Paleolithic intergroup conflict. *Sci. Rep.* **15**, 14857 (2025). 10.1038/s41598-025-94095-x PMC12037905 40295539

[69] Bachechi, L., Fabbri, P. F. & Mallegni, F. An arrow-caused lesion in a Late Upper Palaeolithic human pelvis. *Curr. Anthropol.* **38**, 135–140 (1997).

[70] Henry-Gambier, D. et al. *La Sépulture des enfants de Grimaldi (Baoussé-Roussé, Italie)-Anthropologie et palethnologie funéraire des populations de la fin du Paléolithique supérieur* (Éditions du CTHS, 2001).

[71] Kranioti, E. F., Grigorescu, D. & Harvati, K. Cioclovina fractures: Reply to Soficaru and Trinkaus: Perimortem versus postmortem damage: The recent case of Cioclovina 1, Am. J. Phys. Anthropol. 2020 172, 135–139. *Am. J. Phys. Anthropol.* **174**, 575–579 (2021). 10.1002/ajpa.24209 33368147

[72] Soficaru, A. D. & Trinkaus, E. Perimortem versus postmortem damage: The recent case of Cioclovina 1. *Am. J. Phys. Anthropol.* **172**, 135–139 (2020). 10.1002/ajpa.24045 32150283

[73] Spikins, P. et al. Living to fight another day: The ecological and evolutionary significance of Neanderthal healthcare. *Quat Sci. Rev.* **217**, 98–118 (2019).

[74] Aranguren, B. et al. Wooden tools and fire technology in the early Neanderthal site of Poggetti Vecchi (Italy). *Proc. Natl. Acad. Sci. U S A*. **115**, 2054–2059 (2018). 10.1073/pnas.1716068115 PMC5834685 29432163

[75] Rios-Garaizar, J. et al. A Middle Palaeolithic wooden digging stick from Aranbaltza III, Spain. *PLOS ONE*. **13**, e0195044 (2018). 10.1371/journal.pone.0195044 PMC5874079 29590205

[76] Carbonell, E. & Castro-Curel, Z. Palaeolithic wooden artefacts from the Abric Romaní (Capellades, Barcelona, Spain). *J. Archaeol. Sci.* **19**, 707–719 (1992).

[77] Hardy, B. L. et al. Direct evidence of Neanderthal fibre technology and its cognitive and behavioral implications. *Sci. Rep.* **10**, 4889 (2020). 10.1038/s41598-020-61839-w PMC7145842 32273518

[78] Hardy, K. The use of plants by Neanderthals as food, medicine, and raw materials in Updating Neanderthals (eds Romagnoli, F., Rivals, F. & Benazzi, S.) 145–161 (Academic, (2022).

[79] Harris, E. C. *Principles of Archaeological Stratigraphy* (Elsevier, 2014).

[80] López-Polín, L. et al. A first report on the conservation of the Shanidar A Neanderthal. *Bull Mém Soc. Anthropol. Paris* **38**(S) (2026). 10.4000/15hig

[81] Isensee, F. et al. nninteractive: Redefining 3D promptable segmentation. Preprint at (2025). 10.48550/arXiv.2503.08373

[82] de Vente, C., Venkadesh, K. V., van Ginneken, B., Sánchez, C. I. & Slicernninteractive A 3d slicer extension for nninteractive. *Preprint at.* 10.48550/arXiv.2504.07991 (2025).

[83] Molnar, S. Human tooth wear, tooth function and cultural variability. *Am. J. Phys. Anthropol.* **34**, 175–189 (1971). 10.1002/ajpa.1330340204 5572602

[84] Sala, N. et al. Taphonomic skeletal disturbances in the Sima de los Huesos postcranial remains. *Anat., Rec.* **307**, 2437–2450 (2024). 10.1002/ar.25197 36877151

[85] Bunn, H. BAR International Series,. T in Animals and Archaeology: Hunters and their Prey Vol. 1 (ed. Clutton-Brock, J. & Grigson, C.) 143–148 (1983).

[86] Villa, P. & Mahieu, E. Breakage patterns of human long bones. *J. Hum. Evol.* **21**, 27–48 (1991).

[87] Gómez-Olivencia, A. et al. La Ferrassie 1: New perspectives on a classic Neandertal. *J. Hum. Evol.* **117**, 13–32 (2018). 10.1016/j.jhevol.2017.12.004 29544621

[88] Sala, N., Arsuaga, J. L., Martínez, I. & Gracia-Téllez, A. Breakage patterns in Sima de los Huesos (Atapuerca, Spain) hominin sample. *J. Archaeol. Sci.* **55**, 113–121 (2015).

