# Supplementary material for: Evidence for funerary behaviour and lethal trauma in a new Neanderthal individual from Shanidar Cave
Source: Sci Rep. 2026 Oct 15;16:30042. doi: 10.1038/s41598-026-69193-z (PMC13672083; doi:10.1038/s41598-026-69193-z)
Supplement: Supplementary file 1 [file 41598_2026_69193_MOESM1_ESM.docx]

**Supplementary Methods**

**Evidence for funerary behaviour and lethal trauma in a new Neanderthal individual from Shanidar Cave**

Emma Pomeroy^1^*, Christopher O. Hunt^2^, Lucía López-Polín^3,4,5^, Nohemi Sala^6^, Ana Pantoja Pérez^6^, James Holman^7^, Jessica Twyman^7^, Paul Bennett^7^, Tim Reynolds^8^, Ross Lane^7^, Duncan Pirrie^9^, Graeme Barker^3^.

^1^ Department of Archaeology, University of Cambridge, UK

^2^ School of Biological and Environmental Sciences, Liverpool John Moores University, Liverpool, UK

^3^ McDonald Institute for Archaeological Research, University of Cambridge

^4^ Institut Català de Paleoecologia Humana i Evolució Social, Spain

^5^ Universitat Rovira i Virgili, Spain

^6^ Centro Nacional de Investigación sobre la Evolución Humana, Burgos, Spain

^7^ Canterbury Archaeological Trust, Canterbury, UK

^8^ Birkbeck, University of London, UK

^9^ Helford Geoscience LLP, UK

**Supplementary Methods: Taphonomic alterations**

Bone surface modifications were investigated following established taphonomic standards widely applied in palaeoanthropological research (Sala et al.^1^ and references therein). All skeletal remains were systematically examined macroscopically and microscopically using a binocular stereomicroscope (Nikon SMZ745T) to screen for the presence of modifications acquired during both the biostratinomic and post-burial (fossil-diagenetic) stages.

The assessment included the systematic inspection for carnivore tooth marks (tooth pits, punctures, scores and furrowing)^2-4^, anthropogenic modifications (cut marks, percussion marks and thermal alteration)^5-7^, rodent gnawing^8,9^, weathering^10^, sedimentary abrasion and trampling^11-13^, root etching^14,15^, insect and microbial bioerosion, mineral precipitates (e.g. manganese oxides and carbonate crusts)^16,17^, and chemical dissolution or other diagenetic alterations^15^. The presence or absence of each modification was systematically recorded for every specimen. Whenever diagnostic modifications were identified, their morphology, anatomical distribution and diagnostic features were characterised following the specific methodological criteria established for each modification type in the corresponding references cited above.

Fracture pattern analysis followed the criteria proposed by Bunn⁷⁰ and Villa and Mahieu⁷¹, as applied in recent taphonomic studies of hominin assemblages^1,18-21^. For each specimen, fracture outline (longitudinal, transverse or oblique/curved relative to the long axis of the bone), fracture angle (right or oblique), and fracture edge morphology (smooth or jagged) were recorded. In long bones, shaft circumference completeness (<50%, >50%, or complete circumference) and preserved shaft length (<1/4, ¼-1/2, 1/2–3/4, or >3/4 of the original diaphyseal length) were additionally assessed. The combination of these attributes was used to distinguish fractures produced while bone retained its biomechanical properties (fresh-bone or perimortem fractures) from those generated after collagen loss during post-depositional processes or excavation (dry-bone or postmortem fractures).

**References**

1	Sala, N. *et al.* Taphonomic skeletal disturbances in the Sima de los Huesos postcranial remains. *Anat. Rec.* **307(7)**, 2437-2450 (2024).

2	Sala, N. & Arsuaga, J. L. Regarding beasts and humans: a review of taphonomic works with living carnivores *Quatern. Int.* **466**, 131-140 (2018).

3	Binford, L. R. *Bones: Ancient Men and Modern Myths*. (Academic Press, 1981).

4	Haynes, G. A guide for diferentiating mammalian carnivore taxa responsible for gnaw damage to herbivore limb bones. *Paleobiology* **9**, 164-172 (1983).

5	Saladié, P. *et al.* Intergroup cannibalism in the European Early Pleistocene: The range expansion and imbalance of power hypotheses. *J. Hum. Evol.* **63**, 682-695 (2012).

6	Bello, S. M., Wallduck, R., Dimitrijević, V., Živaljević, I. & Stringer, C. B. Cannibalism versus funerary defleshing and disarticulation after a period of decay: comparisons of bone modifications from four prehistoric sites. *Am. J. Phys. Anthropol.* **161**, 722-743 (2016).

7	Bello, S. M. & Soligo, C. A new method for the quantitative analysis of cutmark micromorphology. *J. Archaeol. Sci.* **35**, 1542-1552 (2008).

8	Haglund, W. D. Rodents and human remains in *Forensic Taphonomy: The Postmortem Fate of Human Remains* (ed. Haglund, W.D. & Sorg M.H.) 405-414 (CRC Press, 1997).

9	Klippel, W. E. & Synstelien, J. A. Rodents as taphonomic agents: bone gnawing by brown rats and gray squirrels. *J. Forensic. Sci.* **52** (2007).

10	Behrensmeyer, A. K. Taphonomic and ecologic information from bone weathering. *Paleobiology* **4**, 150-162 (1978).

11	Domínguez-Rodrigo, M., Juana, S. d., Galán, A. B. & Rodríguez, M. A new protocol to differentiate trampling marks from butchery cut marks. *J. Archaeol. Sci.* **36**, 2643–2654 (2009).

12	Pineda, A. *et al.* Trampling versus cut marks on chemically altered surfaces: an experimental approach and archaeological application at the Barranc de la Boella site (La Canonja, Tarragona, Spain). *J. Archaeol. Sci.* **50**, 84-93 (2014).

13	Behrensmeyer, A. K., Gordon, K. D. & Yanagi, G. T. Trampling as a cause of bone surface damage and pseudo-cutmarks. *Nature* **319**, 768-771 (1986).

14	Macho-Callejo, A., García-Morato, S., Gutiérrez, A., Marin-Monfort, D. & Fernández-Jalvo, Y. Put down roots and find the plant!: preliminary results of root etching and its implications. *Hist. Biol.* **36**, 2502-2510 (2024).

15	Lyman, R. L. *Vertebrate Taphonomy*. (Cambridge University Press, 1994).

16	Marín-Arroyo, A. B. *et al.* Archaeological implications of human-derived manganese coatings: a study of blackened bones in El Mirón Cave, Cantabrian Spain. *J. Archaeol. Sci.* **35**, 801-813 (2008).

17	White, W. B. & Culver, D. C. *Encyclopedia of Caves* (Academic Press, 2012).

18	Sala, N., Arsuaga, J. L., Martínez, I. & Gracia-Téllez, A. Breakage patterns in Sima de los Huesos (Atapuerca, Spain) hominin sample. *J. Archaeol. Sci.* **55**, 113-121 (2015).

19	Sala, N., Pantoja-Pérez, A., Arsuaga, J. L., Pablos, A. & Martínez, I. The Sima de los Huesos crania: Analysis of the cranial breakage patterns. *J. Archaeol. Sci.* **72**, 25-43.

20	Gómez-Olivencia, A. *et al.* La Ferrassie 1: New perspectives on a "classic" Neandertal. *J Hum Evol* **117**, 13-32 (2018).

21	Pantoja-Pérez, A., Martín-Francés, L., May, H., Hershkovitz, I. & Sala, N. A taphonomic reassessment of Qafzeh 25 and its implications for violence, health and funerary practices. *Sci. Rep.* **16***,* 13908 (2026).
