# Supplementary material for: Evidence for funerary behaviour and lethal trauma in a new Neanderthal individual from Shanidar Cave
Source: Sci Rep. 2026 Oct 15;16:30042. doi: 10.1038/s41598-026-69193-z (PMC13672083; doi:10.1038/s41598-026-69193-z)
Supplement: Supplementary file 2 [file 41598_2026_69193_MOESM4_ESM.docx]

**Supplementary Information**

Video S1. 3D rendering of the scapula perforation showing the location in the underlying sediment of fragments displaced from the perforation in a dorsal direction.
